# A useful technique of starting internal limiting membrane peeling from the edge of the internal limiting membrane defect in epiretinal membrane surgery

**DOI:** 10.1002/ccr3.7279

**Published:** 2023-04-22

**Authors:** Hirofumi Sasajima, Masahiro Zako

**Affiliations:** ^1^ Department of Ophthalmology Shinseikai Toyama Hospital Imizu Japan; ^2^ Yamada Eye Clinic Nagano Japan; ^3^ Department of Ophthalmology Asai Hospital Seto Japan

**Keywords:** epiretinal membrane, internal limiting membrane, nerve fiber layer, optical coherence tomography angiography, peeling

## Abstract

Clinicians should be aware that internal limiting membrane (ILM) defects may occur concurrently with epiretinal membrane, and starting ILM peeling at the ILM defect margin may be useful in such cases.

**Abstract:**

We describe a useful surgical technique for the treatment of idiopathic epiretinal membrane with concurrent internal limiting membrane (ILM) defect, in which ILM peeling was started from the ILM defect margin. A dissociated optic nerve fiber layer‐like appearance on fundus examination and optical coherence tomography may suggest an ILM defect.

## 1 INTRODUCTION

Epiretinal membranes (ERMs) are the most common fibrocellular proliferations on the internal limiting membrane (ILM).^1^, ^2^ Pars plana vitrectomy (PPV) is the only treatment for ERM,^3^, ^4^ and ILM peeling is often performed during ERM surgery since recurrence of ERM is less likely with ILM peeling than without it.^5^ It has also been reported that the visual prognosis of ERM surgery may be affected by whether or not ERM recurs.^6^ Therefore, ILM peeling may be an important technique for determining visual prognosis after ERM surgery.

Previous studies reported that ILM defects are sometimes observed in eyes with ERM.^7^, ^8^, ^9^ Feldman et al. reported that a spontaneous ILM rip was observed intraoperatively using infracyanine green before ILM peeling in 10 of 44 (22.7%) eyes during ERM surgery.^7^ Another recent study reported two cases of idiopathic ERM with concurrent ILM defect and clearly depicted ILM defect margins.^9^ We speculate that starting ILM peeling from the ILM defect margin could be a useful technique because it contributes to avoiding intraoperative damage to the retinal nerve fiber layer (RNFL) in the area of the ILM defect.

Herein, we present a useful technique in which ILM peeling was started from the ILM defect margin during surgical treatment of ERM with concurrent ILM defect.

## 2 CASE REPORT

**FIGURE 1:**
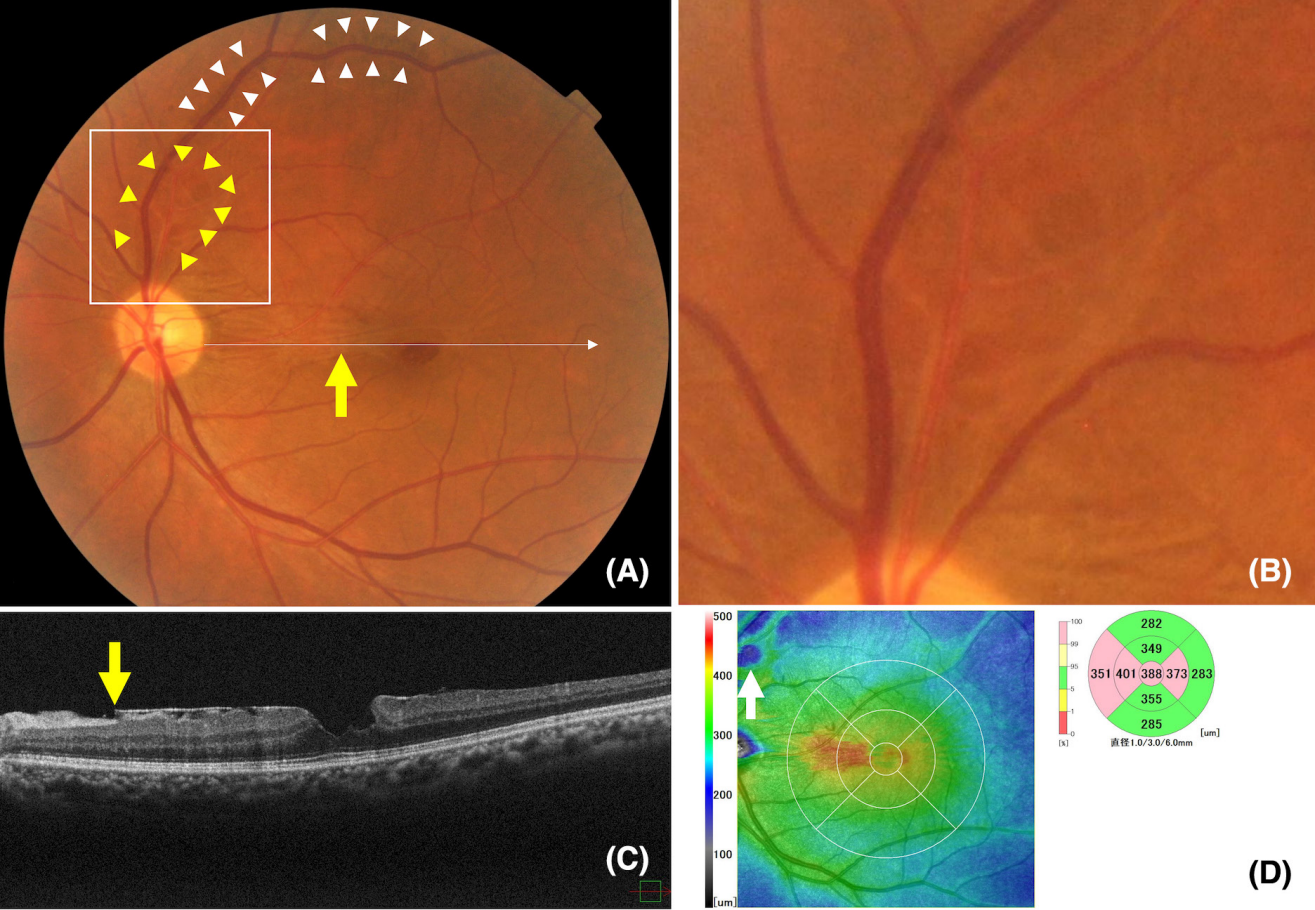
Preoperative findings in the left eye. (A) Color fundus photograph shows an ERM (yellow arrow), paravascular inner retinal defects along the superior temporal arcade vein (white arrow heads), and a DONFL‐like appearance (within yellow arrow heads in the white square). (B) Magnified image of the white square in (A) shows a DONFL‐like appearance. (C) Horizontal OCT image of the arrow in (A) shows an ERM. ERM tear is observed at the arrow site. (D) OCT map shows focal thinning area (arrow) corresponding to the area with a DONFL‐like appearance in (A). DONFL, dissociated optic nerve fiber layer; ERM, epiretinal membrane; OCT, optical coherence tomography.

A 58‐year‐old healthy Japanese woman was referred to Shinseikai Toyama Hospital for treatment of cataract and ERM in the left eye. At the initial visit, she presented with blurred vision and metamorphopsia in the left eye. Upon examination, the best‐corrected visual acuity (BCVA) was 20/25 in the right eye and 10/20 in the left eye. The intraocular pressure was 10 mmHg in both eyes, and the axial length was 22.96 and 22.82 mm in the right and left eye, respectively. Moderate nuclear cataracts were observed in both eyes. Fundus examination revealed an ERM, paravascular inner‐retinal defects in the left eye that appeared as caterpillar‐shaped dark areas along the superior temporal arcade vein (Figure 1A), and a dissociated optic nerve fiber layer (DONFL)‐like appearance near the superior temporal arcade vein (Figure 1A,B). Optical coherence tomography (OCT, RS‐3000, Nidek Co., Ltd.) showed no abnormality in the right eye; however, an ERM and ERM tear was observed in the left eye (Figure 1C). The central macular thickness was 270 and 388 μm in the right and left eye, respectively. Moreover, the OCT map showed no abnormality in the right eye but showed an area of focal thinning near the superior temporal arcade vein in the left eye (Figure 1D).

**FIGURE 2:**
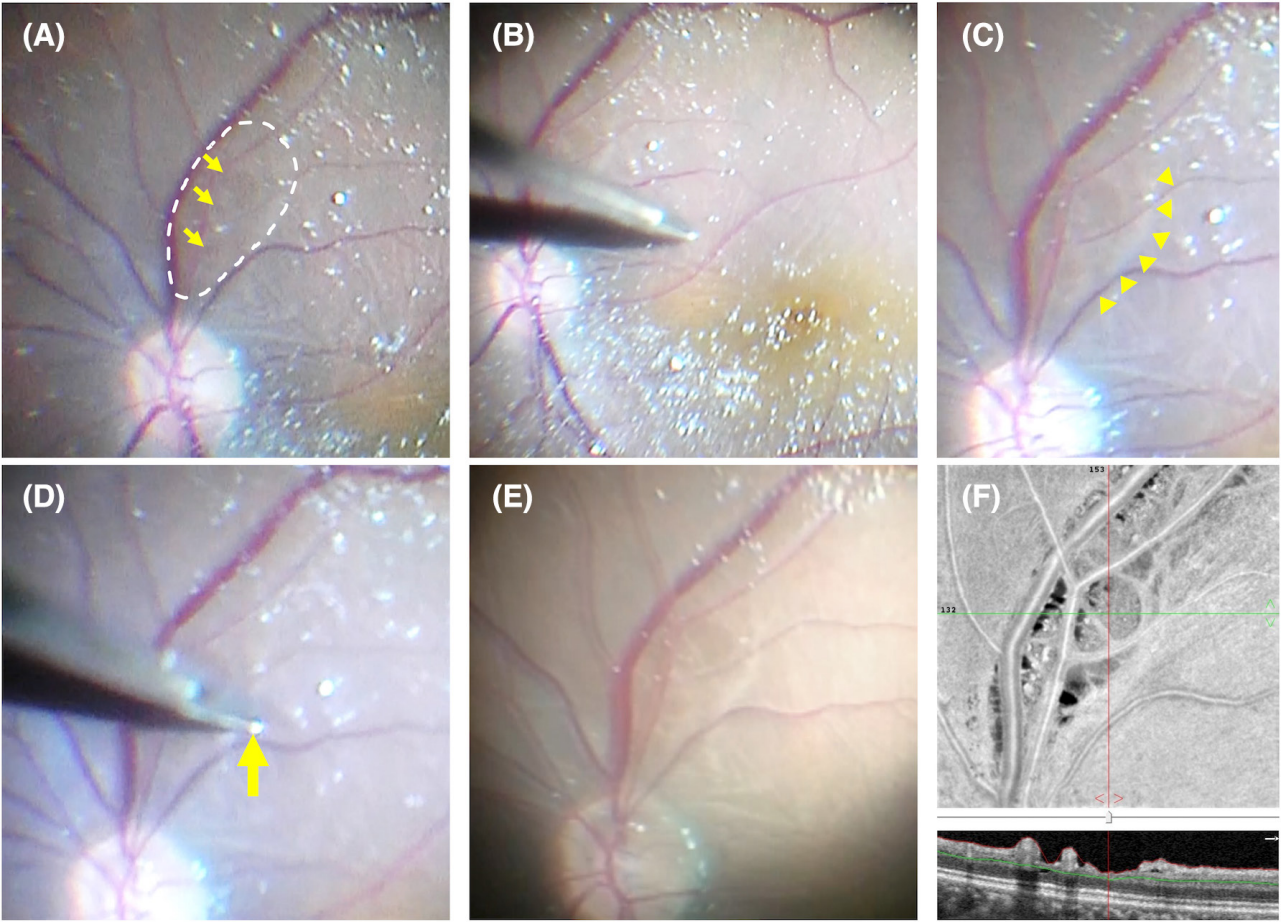
Intraoperative and postoperative findings in the left eye. (A) Image after spraying triamcinolone acetonide and BBG prior to ERM and ILM peeling. A DONFL‐like appearance (arrows) is observed and the area (within the white dotted circle) does not show ERM and BBG staining. (B) Image at the initiation of ERM peeling. (C) Image after ERM peeling. The area with a DONFL‐like appearance is not stained with BBG, and the ILM defect margin is clearly delineated (arrowheads). (D) Image at the initiation of ILM peeling from the margin of the ILM defect (arrow). (E) Image after ILM peeling and spraying of BBG. The area with a DONFL‐like appearance is not stained with BBG. (F) Postoperative OCT en‐face image at the superficial level. DONFL‐like low‐brightness spots are observed in the area where an ILM defect was suspected intraoperatively. OCT B scan image shows an inner retinal layer defect. BBG, Brilliant Blue G; DONFL, dissociated optic nerve fiber layer; ERM, epiretinal membrane; ILM, internal limiting membrane; OCT, optical coherence tomography.

The patient underwent phacoemulsification with PPV combined with ERM and ILM peeling in the left eye. Core vitrectomy was performed after phacoemulsification and intraocular lens implantation. During core vitrectomy, we confirmed that posterior vitreous detachment had already occurred by spraying triamcinolone acetonide. After core vitrectomy, Brilliant Blue G (BBG) was gently sprayed onto the macula before ERM peeling. An area with a DONFL‐like appearance that was not stained with BBG and without ERM was observed near the superior temporal arcade vein, corresponding to the area of focal thinning on the preoperative OCT map (Figure 2A).

The findings suggested an ILM defect, as described in a previous report.^6^ Therefore, ERM peeling was started from another site (Figure 2B). After ERM peeling, BBG was gently sprayed again onto the macula before ILM peeling. The area with a DONFL‐like appearance was not stained with BBG, but the ILM defect margin was clearly delineated (Figure 2C). ILM peeling was started from the ILM defect margin (Figure 2D) and was completed without RNFL damage. BBG was sprayed again on the area with a DONFL‐like appearance, and the absence of BBG staining of the remaining tissue was reconfirmed (Figure 2E).

Postoperative OCT en‐face images at the superficial level (RTVue XR Avanti, Optovue, Inc.) revealed DONFL‐like low‐brightness spots (Figure 2F), which corresponded to the area where an ILM defect was suspected intraoperatively. Four months after surgery, no ERM was observed on OCT images of the left eye. Metamorphopsia was completely resolved, and the BCVA improved to 20/25 in the left eye.

## 3 DISCUSSION

We presented a useful technique for the surgical treatment of ERM with concurrent ILM defects, in which ILM peeling was initiated from the ILM defect margin to avoid RNFL damage. Clinicians occasionally encounter cases with concurrent ERM and ILM defects. We believe that this technique can contribute to the relatively easy initiation of ILM peeling without damaging the RNFL during surgical treatment of ERM with ILM defects. In our patient, the ILM at the defect margin had an arcuate shape and was well‐stained with BBG. This shape was similar to that of the ILM at the defect margins in a previous study.^9^ Therefore, this shape may be helpful in identifying ILM defect margins.

In a previous study, areas with DONFL‐like low‐brightness spots on OCT en‐face images were useful in predicting ILM defects.^9^ In this case, DONFL‐like low‐brightness spots were also observed in the ILM defect area. Furthermore, in this case, the area with focal thinning on the preoperative OCT map corresponded to the ILM defect location. Therefore, OCT maps may also be useful in predicting the location of ILM defects.

Although the most frequent site of ILM defects in patients with ERM has not been determined, we speculate that ILM defects are more likely to occur along the temporal arcade vessels toward the posterior pole due to afferent contraction of the ERM because the arcade vessels have strong adhesion with the posterior hyaloid.^10^, ^11^ Further studies are needed to investigate the predominant site of ILM defects in patients with ERM.

The postoperative outcome of ERM with ILM defect was evaluated in a recent study.^8^ The study reported that a spontaneous tear in the ILM at the periphery of the ERM was found in 22.4% of eyes. A spontaneous tear was associated with the severity of the ERM but not with the visual outcome.^8^ In this case, the BCVA of the left eye improved from 10/20 to 20/25. Further studies are warranted to elucidate the efficacy of this technique in avoiding RNFL damage during surgical treatment of ERM with ILM defects.

This case report had some limitations. First, this is the only case showing the usefulness of starting ILM peeling from the margin of the ILM defect. Second, preoperative OCT en‐face imaging was not performed in this case. OCT en‐face images may be useful in identifying ILM defect areas and margins.^9^ Future studies need to investigate the usefulness of preoperative OCT en‐face imaging.

## 4 CONCLUSIONS

Clinicians should be aware that ILM defects may occur concurrently with ERM, and starting ILM peeling at the ILM defect margin may be useful in such cases.

## AUTHOR CONTRIBUTIONS

**Hirofumi Sasajima:** Conceptualization; data curation; investigation; methodology; project administration; supervision; validation; visualization; writing – original draft. **Masahiro Zako:** Supervision; writing – review and editing.

## FUNDING INFORMATION

No funding was received for this article.

## CONFLICT OF INTEREST STATEMENT

The authors have no conflicts of interest to declare.

## ETHICS STATEMENT

The Ethics Committee of Shinseikai Toyama Hospital waived the need for approval of this report, as it involved a retrospective review of medical records. This study adhered to the tenets of the Declaration of Helsinki, 1964. Written informed consent was obtained from the patient for the publication of this case report and accompanying images.

## CONSENT

Written informed consent was obtained from the patient to publish this report in accordance with the journal's patient consent policy.

## Data Availability

Data sharing not applicable to this article as no datasets were generated or analysed during the current study.
